# Exposure to passive heat and cold stress differentially modulates cerebrovascular-CO_2_ responsiveness

**DOI:** 10.1152/japplphysiol.00494.2023

**Published:** 2023-11-16

**Authors:** Bethany D. Skinner, Rebekah A. I. Lucas, Samuel J. E. Lucas

**Affiliations:** ^1^School of Sport, Exercise and Rehabilitation Sciences, University of Birmingham, Birmingham, United Kingdom; ^2^Centre for Human Brain Health, University of Birmingham, Birmingham, United Kingdom

**Keywords:** cerebral blood flow, cerebrovascular function, cold stress, heat stress

## Abstract

Heat and cold stress influence cerebral blood flow (CBF) regulatory factors (e.g., arterial CO_2_ partial pressure). However, it is unclear whether the CBF response to a CO_2_ stimulus (i.e., cerebrovascular-CO_2_ responsiveness) is maintained under different thermal conditions. This study aimed to compare cerebrovascular-CO_2_ responsiveness between normothermia, passive heat, and cold stress conditions. Sixteen participants (8 females; 25 ± 7 yr) completed two experimental sessions (randomized) comprising normothermic and either passive heat or cold stress conditions. Middle and posterior cerebral artery velocity (MCA*v*, PCA*v*) were measured during rest, hypercapnia (5% CO_2_ inhalation), and hypocapnia (voluntary hyperventilation to an end-tidal CO_2_ of 30 mmHg). The linear slope of the cerebral blood velocity (CB*v*) response to changing end-tidal CO_2_ was calculated to measure cerebrovascular-CO_2_ responsiveness, and cerebrovascular conductance (CVC) was used to examine responsiveness independent of blood pressure. CB*v*-CVC-CO_2_ responsiveness to hypocapnia was greater during heat stress compared with cold stress (MCA: +0.05 ± 0.08 cm/s/mmHg/mmHg, *P* = 0.04; PCA: +0.02 ± 0.02 cm/s/mmHg/mmHg, *P* = 0.002). CB*v*-CO_2_ responsiveness to hypercapnia decreased during heat stress (MCA: −0.67 ± 0.89 cm/s/mmHg, *P* = 0.02; PCA: −0.64 ± 0.62 cm/s/mmHg; *P* = 0.01) and increased during cold stress (MCA: +0.98 ± 1.33 cm/s/mmHg, *P* = 0.03; PCA: +1.00 ± 0.82 cm/s/mmHg; *P* = 0.01) compared with normothermia. However, CB*v*-CVC-CO_2_ responsiveness to hypercapnia was not different between thermal conditions (*P* > 0.08). Overall, passive heat, but not cold, stress challenges the maintenance of cerebral perfusion. A greater cerebrovascular responsiveness to hypocapnia during heat stress likely reduces an already impaired cerebrovascular reserve capacity and may contribute to adverse events (e.g., syncope).

**NEW & NOTEWORTHY** This study demonstrates that thermoregulatory-driven perfusion pressure changes, from either cold or heat stress, impact cerebrovascular responsiveness to hypercapnia. Compared with cold stress, heat stress poses a greater challenge to the maintenance of cerebral perfusion during hypocapnia, challenging cerebrovascular reserve capacity while increasing cerebrovascular-CO_2_ responsiveness. This likely exacerbates cerebral hypoperfusion during heat stress since hyperthermia-induced hyperventilation results in hypocapnia. No regional differences in middle and posterior cerebral artery responsiveness were found with thermal stress.

## Graphical abstract

**Figure:**
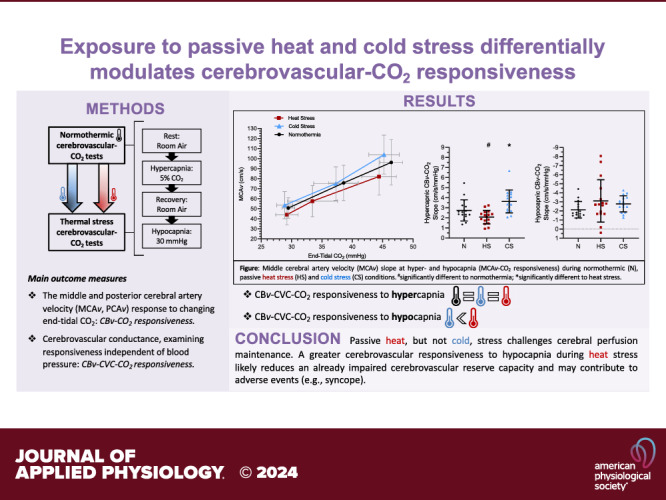

## INTRODUCTION

Exposure to thermal stressors (i.e., heat and cold stress) poses a substantial challenge to the maintenance of cerebral perfusion. The brain has a high energy demand yet very limited storage capacity, and, therefore, cerebral blood flow (CBF) is tightly regulated to maintain a sufficient supply of substrates and to preserve optimal function (1). Control of CBF is dynamic, adjusting to changes in perfusion pressure, the brain’s metabolic activity, humoral factors, autonomic nerve activity, and with particular sensitivity to changes in arterial CO_2_ partial pressure (${\text{Pa}}_{{\text{CO}}_{\text{2}}}$) (2). Heat and cold stress influence, both directly and indirectly, most of these regulatory factors involved in the control of CBF, with CBF disruption associated with events such as syncope or impaired cognitive function. Subsequently, maintenance of CBF is of particular importance for performance or survival across the full range of thermal environments.

Passive heat stress causes a reduction in CBF, with a 1°C increase in body core temperature (T_C_) reducing CBF by 10%–15% (3), whereas a 2°C increase in T_C_ reduces CBF by ∼30% (4). These hyperthermia-induced reductions in CBF appear to be predominantly driven by changes in ${\text{Pa}}_{{\text{CO}}_{\text{2}}}$ as a result of hyperventilation-induced hypocapnia (5–7). Although ${\text{Pa}}_{{\text{CO}}_{\text{2}}}$ is a key regulator of CBF during heat stress, it is unclear whether the CBF response to a CO_2_ stimulus (i.e., cerebrovascular responsiveness to CO_2_) is maintained under different thermal conditions. Previous studies have shown cerebrovascular-CO_2_ responsiveness to increase (8, 9), decrease (10), or remain similar (11) during passive heat stress when compared with normothermic conditions. These conflicting outcomes may be the result of different insonated vessels (e.g., middle cerebral artery, internal carotid artery), the CO_2_ stimulus range examined (e.g., only hypercapnia, both hyper- and hypocapnia), or the strength of the heat stress stimulus used (e.g., +0.5°C, +1.5°C). In addition, performing a hypocapnic challenge before hypercapnia has since been shown to blunt the subsequent hypercapnic response in normothermia (12), such that previous reports may be confounded by this order effect (11). Therefore, how cerebrovascular-CO_2_ responsiveness changes with passive heat stress remains unclear.

The effect of passive cold stress on CBF is less well documented. The majority of previous research has been conducted using cold-water immersion, reporting that CBF is reduced (9, 13), and that this reduction is primarily mediated by thermally induced hyperventilation [i.e., the cold shock response (14)]. Conversely, passive cold exposure appears to result in no substantial change in CBF when compared with normothermia (15). To the best of our knowledge, only one study has examined cerebrovascular-CO_2_ responsiveness to hypocapnia during cold exposure, showing a tendency for increased responsiveness to hypocapnia during cold-water immersion when compared with normothermia [*P* = 0.05 (9)]. Improved understanding of the mechanisms governing CBF during different thermal stressors has important implications for physical and/or cognitive performance or survival in extreme thermal environments.

Potential regional differences in CBF regulation have implications for CBF control during thermal stress, with the anterior and posterior circulation supplying brain regions vital for cognitive function and consciousness, respectively. Previous research has reported that cerebrovascular-CO_2_ responsiveness differs between CBF vessels under normothermic conditions, although both a lower (16) and greater (17) responsiveness has been reported in the posterior circulation when compared with the anterior circulation. A formal comparison of cerebrovascular-CO_2_ responsiveness in intracranial arteries of the anterior and posterior circulations during perturbations in core temperature has not been examined.

Subsequently, the primary aim of this study was to compare cerebrovascular-CO_2_ responsiveness between normothermic, passive heat stress, and passive cold stress conditions. A secondary aim of this study was to compare cerebrovascular-CO_2_ responsiveness in the anterior and posterior circulations within normothermic, passive heat stress, and passive cold stress conditions. Previous studies indicate that cerebovascular-CO_2_ responsiveness of the intracranial arteries is greater during thermal stress compared with normothermia. Evidence of regional differences in cerebrovascular-CO_2_ responsiveness during thermal stress is currently limited, but preservation of the posterior circulation might be expected to maintain CBF to brain regions vital for consciousness. As such, it was hypothesized that cerebrovascular-CO_2_ responsiveness would be *1*) greater during passive heat and passive cold stress compared with normothermia and *2*) greater in the posterior circulation compared with the anterior circulation during thermal stress.

## MATERIALS AND METHODS

The study was approved by the University of Birmingham Ethics Committee (ERN_15–1179) and all participants gave written, informed consent before enrolling in the study, in adherence with the Declaration of Helsinki.

### Study Design and Protocol

**Figure 1.**
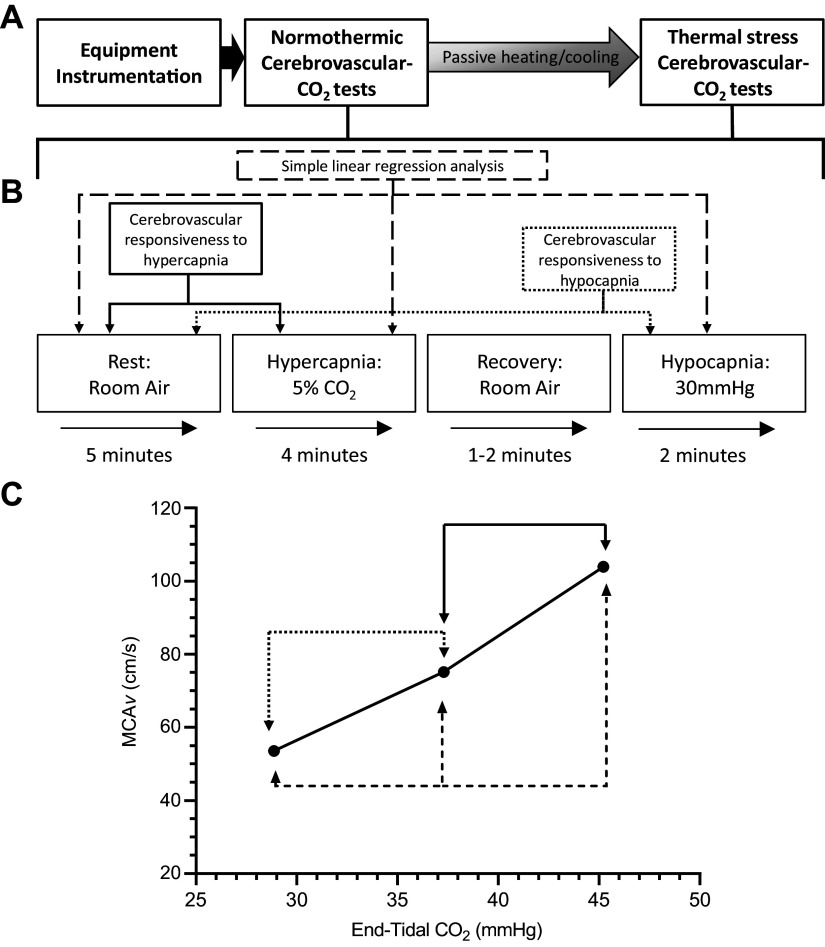
Schematic of the study protocol. *A*: an overview of the experimental session. *B*: the cerebrovascular-CO_2_ responsiveness tests protocol (performed twice, during normothermia and passive thermal stress conditions). *C*: an example graph of the cerebrovascular responsiveness slope. Solid and dotted arrows indicate points of data extraction to calculate cerebrovascular responsiveness to hypercapnia and hypocapnia, respectively. Dashed arrows indicate points of data extraction used in simple linear regression analysis.

Sixteen participants [8 females; age: 25 ± 7 yr; body mass index (BMI) 23.8 ± 3.7 kg/m^2^] took part in this study. Participants were required to attend the laboratory on three occasions. During the first visit, written informed consent was obtained and a general health questionnaire was completed to ensure inclusion/exclusion criteria were met. All participants were healthy and free of any known cardiovascular, neurological, or metabolic diseases. Female participants were not taking any hormonal contraceptive medication and attended experimental testing sessions during the early follicular phase of the menstrual cycle (*days 1*–*7*). Following successful screening, participants completed a familiarization session of the cerebrovascular-CO_2_ responsiveness protocol. They were asked to lie in a supine position for a minimum of 20 min before beginning the cerebrovascular-CO_2_ responsiveness tests (Fig. 1). A 5-min period of resting data was collected with inhalation of room air, followed by hypercapnic (4 min) and hypocapnic stages (∼2 min). Hypercapnia was induced by inhalation of a 5% CO_2_ gas mixture (21% O_2_, balanced N_2_), and hypocapnia was induced by guided voluntary hyperventilation to an end-tidal CO_2_ target of 30 mmHg. The order of the cerebrovascular-CO_2_ responsiveness tests was kept consistent, as hypocapnia has been shown to blunt subsequent cerebrovascular responses to hypercapnia (12).

On arrival at an experimental testing session (two repeat visits, a minimum of 48 h apart), baseline body mass was measured and participants were fitted with thermistors to measure core and skin temperature (see *Equipment and Outcome Measures*). Participants dressed in a water-perfused tube-lined suit (Med-Eng, Ottawa, Canada) covering the entire body, excluding the head, face, hands, and feet. Thermoneutral water (34°C) perfused the suit, while participants lay supine for a minimum of 20 min during which they were instrumented for measures of cerebro- and cardiovascular function (see *Equipment and Outcome Measures*). Normothermic cerebrovascular-CO_2_ responsiveness tests were then performed before participants underwent either a passive heating or passive cooling protocol, the order of which was randomized. Passive heat stress was induced by circulation of ∼49°C water until an increase in T_C_ of 1°C, after which circulating water temperature was lowered by ∼2°C to prevent further increases in T_C_. Passive cooling was induced by circulation of ∼14°C water until either the onset of involuntary and uncontrollable shivering or a decrease in T_C_ of 0.5°C was observed. This decrease was taken from the peak core temperature attained following the transient rise in T_C_ from the initial redistribution of cardiac output after the initiation of cold stress. Water temperature was increased or decreased as necessary to maintain a constant core temperature, while the cerebrovascular-CO_2_ responsiveness tests were repeated under thermal stress.

### Equipment and Outcome Measures

Beat-to-beat middle and posterior cerebral artery blood velocities (MCA*v*, PCA*v*) were assessed using transcranial Doppler (TCD; Doppler-Box X, DWL, Compumedics Ltd, Germany) with a 2-MHz probe placed over each temporal window. Ultrasound gel was placed on the probes and held in place with a headset. Where possible, the right MCA and left PCA were insonated; however, the PCA could not be positively identified in four participants (3 females, 1 male).

T_C_ was measured using a rectal thermistor (General Purpose Temperature Probe 400TM, Mon-a-therm, Covidien, Mansfield, MA), with participants instructed to insert the thermistor to a depth of 10 cm past the external anal sphincter. Mean skin temperature (T_Skin_) was measured by the weighted average of four thermistors attached to the skin of the calf, thigh, bicep, and chest (Grant EUS-U, Grant Instruments Ltd., Cambridge, United Kingdom). Both skin and core temperatures were logged and displayed in real time (Grant 2020 Series Squirrel Data Logger, Grant Instruments Ltd.).

Beat-to-beat blood pressure was measured using a finger cuff on the middle finger on the left hand (Portapres, Finapres, Medical System BV, The Netherlands), and heart rate was measured using a 3-lead electrocardiogram. Participants wore a facemask covering the nose and mouth and held in place using a head strap (7450 Mask, Hans Rudolph Inc, Kansas), connected to a two-way, nonrebreathing Y-shaped valve (2730 valve, Hans Rudolph). Partial pressure of end-tidal CO_2_ was sampled from the valve on a breath-by-breath basis, and a heated pneumotachograph was fitted to measure ventilation and respiratory rate (3818 Series, Hans Rudolph).

### Data Analysis

Data from 60 s of the resting period and 30 s of the hypercapnic stage [from 2:30 to 3:00 min; (18)] and 20–30 s of data at the target end-tidal CO_2_ ($\text{P}{\text{ET}}_{{\text{CO}}_{2}}$) during the hypocapnic stage were extracted and used in the statistical analyses. The linear slope of the cerebral blood velocity (CB*v*) response to changes in $\text{P}{\text{ET}}_{{\text{CO}}_{2}}$ was calculated to give an estimation of cerebrovascular-CO_2_ responsiveness in the MCA and PCA (MCA*v*-CO_2_ responsiveness; PCA*v*-CO_2_ responsiveness). Cerebrovascular responsiveness to hypercapnia and hypocapnia was determined by the slope of the CB*v* response from rest to 5% CO_2_ inhalation and rest to 30 mmHg $\text{P}{\text{ET}}_{{\text{CO}}_{2}}$, respectively (Fig. 1). For comparisons between vessels, the hyper- and hypocapnic slope of the CB*v* response to CO_2_ was calculated relative to room air inhalation (%CB*v*/mmHg) to account for differences in absolute velocity between vessels at rest (17). An index of cerebrovascular conductance (CVC) was calculated from the ratio of CB*v* to mean arterial pressure (MAP), allowing examination of cerebrovascular-CO_2_ responsiveness independent of blood pressure. An estimation of CVC to CO_2_ was expressed as the change in CVC per mmHg change in $\text{P}{\text{ET}}_{{\text{CO}}_{2}}$ (MCA*v*-CVC-CO_2_ responsiveness and PCA*v*-CVC-CO_2_ responsiveness). Finally, the pulsatility index in the MCA and PCA (MCA*v*-PI and PCA*v*-PI) was calculated as (systolic CB*v* – diastolic CB*v*)/mean CB*v*.

### Statistical Analysis

Statistical analysis was performed using GraphPad Prism software (Version 8.0.0, GraphPad Software, San Diego, CA). Baseline and cerebrovascular-CO_2_ responsiveness outcomes from the two normothermic trials (i.e., preheat and precold stress) were compared using paired *t* tests. Since data were not found to be significantly different, outcomes for the two normothermic trials were averaged. To compare between thermal conditions (i.e., normothermia vs. heat stress vs. cold stress), one-way repeated-measures ANOVAs were used for all outcome measures, reported as [*F*(between groups degrees of freedom, within groups degrees of freedom) = [*F* value], *P* = [*P* value]]. PCA outcomes were excluded for one participant during the cold stress trial due to drop out of the TCD signal, and subsequently, a mixed-effects model was used to account for missing values. Simple linear regressions were performed on cerebrovascular-CO_2_ responsiveness outcomes to obtain a best-fit value of the CB*v* response across the CO_2_ range (i.e., spanning both the hypo- and hypocapnic response; Fig. 1). The line of best fit is reported as *y* = b*x* + a, where b is the slope of the line and a is the *y*-intercept when *x* = 0. The slope and *y*-intercept values were also compared between thermal conditions. To examine regional differences in cerebrovascular-CO_2_ responsiveness, paired *t* tests were used to compare MCA and PCA outcomes within each thermal condition, reported as [*t*(degrees of freedom) = [*t* value], *P* = [*P* value]]. Data are presented as means ± SD. Statistical significance was based on an α level of 0.05.

## RESULTS

Sixteen participants were enrolled in the study and completed both experimental testing sessions. The PCA could not be insonated in four participants and so MCA*v* and PCA*v* responsiveness outcome measures have data from 16 and 12 participants, respectively.

### Resting Measures

Resting responses can be found in Table 1. T_C_ and T_Skin_ differed between thermal conditions [*F*(2,24) = 73.62 and *F*(2,24) = 478.6, respectively; *P* < 0.001]. T_C_ and T_Skin_ were elevated during heat stress when compared with normothermia (+1.3 ± 0.3°C and +4.1 ± 1.1°C, respectively; *P* < 0.001). During cold stress, T_Skin_ was lower when compared with normothermia (−8.6 ± 1.7°C; *P* < 0.001), whereas T_C_ remained similar (*P* = 0.69). T_C_ and T_Skin_ were elevated during heat stress when compared with cold stress (+1.2 ± 0.6°C and +12.7 ± 1.9°C; *P* < 0.001). Heart rate differed between thermal conditions [*F*(2,24) =127.5, *P* < 0.001], increasing during heat stress when compared with normothermia (+33 ± 11 beats/min; *P* < 0.001) but remaining similar between cold stress and normothermia (*P* = 0.79). During heat stress, heart rate increased when compared with cold stress (+34 ± 11 beats/min; *P* < 0.001). Mean arterial pressure (MAP) differed between thermal conditions [*F*(2,28) = 85.88, *P* < 0.001], with heat stress decreasing and cold stress increasing MAP when compared with normothermia (−17 ± 11 mmHg and +18 ± 9 mmHg, respectively; *P* < 0.001). Cold stress increased MAP when compared with heat stress (+35 ± 11 mmHg; *P* < 0.001). Ventilation was different between thermal conditions [*F*(1,21) = 12.58, *P* < 0.001]. Although on average ventilation was higher during heat stress when compared with normothermia this did not reach statistical significance (*P* = 0.07). Cold stress increased ventilation compared with normothermia (+5.6 ± 3.2 L/min; *P* < 0.001), but was similar between heat stress and cold stress (*P* = 0.11). Tidal volume differed between thermal conditions [*F*(1,17) = 17.68, *P* < 0.001], with tidal volume remaining similar between heat stress and normothermia (*P* = 0.95) but was elevated during cold stress compared with both normothermia (+0.4 ± 0.2 L; *P* < 0.001) and heat stress (+0.4 ± 0.5 L; *P* = 0.008). Breathing frequency was different between thermal conditions [*F*(1,19) = 5.99, *P* < 0.002], with breathing frequency higher during heat stress compared with normothermia (+5 ± 4 breaths/min; *P* < 0.001). Breathing frequency remained similar between cold stress and both normothermia (*P* = 0.70) and heat stress (*P* = 0.16) conditions. End-tidal CO_2_ differed between thermal conditions [*F*(2,24) = 12.02, *P* < 0.001], decreasing during heat stress when compared with normothermia (−5.1 ± 4.0 mmHg; *P* < 0.001). During cold stress, end-tidal CO_2_ was similar to normothermia (*P* = 0.35), but greater when compared with heat stress (+3.8 ± 5.3 mmHg; *P* = 0.03).

**Table 1.** Resting thermoregulatory, cardiovascular, respiratory, and cerebrovascular responses during normothermia, passive heat stress, and passive cold stress (n = 16)

	Normothermia	Heat Stress	Cold Stress
T_C_, °C	36.8 ± 0.2	38.1 ± 0.4#	36.9 ± 0.4*
T_Skin_, °C	33.4 ± 0.8	37.5 ± 1.2#	24.9 ± 1.7#*
Heart rate, beats/min	65 ± 11	98 ± 10#	64 ± 11*
Mean arterial pressure, mmHg	82 ± 9	66 ± 10#	100 ± 7 #*
Ventilation, L/min	6.5 ± 1.4	9.0 ± 3.7	12.1 ± 3.7#
Tidal volume, L	0.50 ± 0.15	0.53 ± 0.21	0.94 ± 0.32#*
Respiratory frequency, breaths/min	15 ± 3	20 ± 6#	15 ± 5
End-tidal CO_2_, mmHg	38.6 ± 2.4	33.5 ± 4.1#	37.3 ± 3.7*
MCA*v*, cm/s	76 ± 18	59 ± 16#	75 ± 17*
PCA*v*, cm/s	54 ± 8	42 ± 13#	53 ± 10*
MCA*v*-PI	0.79 ± 0.12	1.12 ± 0.21#	0.81 ± 0.13*
PCA*v*-PI	0.75 ± 0.10	1.01 ± 0.21#	0.74 ± 0.70*
MCA*v*-CVC, cm/s/mmHg	0.92 ± 0.17	0.89 ± 0.24	0.75 ± 0.18#*
PCA*v*-CVC, cm/s/mmHg	0.66 ± 0.11	0.64 ± 0.20	0.53 ± 0.13#

Values are represented as means ± SD; *n* − 4 participants for PCA outcome variables in normothermic and heat stress conditions, *n* − 5 participants for PCA outcome variables in cold stress condition. Data analyzed using a one-way repeated measures ANOVA or mixed-effects model. #Significantly different from normothermia; *significantly different from heat stress. MCA*v*, middle cerebral artery velocity; MCA*v*-CVC, middle cerebral artery conductance; PCA*v*, posterior cerebral artery velocity; PCA*v*-CVC, posterior cerebral artery conductance; T_C_, core body temperature; T_Skin_, mean skin temperature.

MCA*v* and PCA*v* differed between thermal conditions [*F*(2,27) = 16.86 and *F*(1,15) = 13.71, respectively; *P* < 0.001]. During heat stress, MCA*v* and PCA*v* decreased when compared with normothermia (MCA*v*: –17 ± 14 cm/s, *P* < 0.001; PCA*v*: –11 ± 9 cm/s, *P* = 0.004). MCA*v* and PCA*v* remained similar between cold stress and normothermia (*P* > 0.88) but were higher during cold stress when compared with heat stress (MCA*v*: +16 ± 15 cm/s, *P* = 0.002; PCA*v*: +10 ± 10 cm/s, *P* = 0.01). MCA*v*-CVC and PCA*v*-CVC differed between thermal conditions [*F*(2, 22) = 9.72, *P* = 0.002 and *F*(1,14) = 6.04, *P* = 0.02, respectively]. MCA*v*-CVC and PCA*v*-CVC remained similar between heat stress and normothermia (*P* > 0.83) but decreased during cold stress when compared with normothermia (MCA*v*: −0.16 ± 0.10 cm/s/mmHg; PCA*v*: −0.13 ± 0.07 cm/s/mmHg; both *P* < 0.001). MCA*v*-CVC decreased during cold stress compared with heat stress (−0.15 ± 0.20 cm/s; *P* = 0.02), whereas PCA*v*-CVC did not differ between cold and heat stress (*P* = 0.11). MCA*v*-PI and PCA*v*-PI were significantly different between thermal conditions [*F*(1,18) = 58.89 and *F*(1,13) = 30.37, respectively; *P* < 0.001]. During heat stress, MCA*v*-PI and PCA*v*-PI increased when compared with normothermia (+0.33 ± 0.15 and +0.27 ± 0.15, respectively; both *P* < 0.001). Both MCA*v*-PI and PCA*v*-PI remained similar between normothermia and cold stress conditions (*P* > 0.50) but were reduced during heat stress when compared with cold stress (+0.31 ± 0.17 and +0.29 ± 0.18 for MCA*v*-PI and PCA*v*-PI, respectively; both *P* < 0.001).

### Cerebrovascular-CO_2_ Responsiveness during Thermal Stress

#### Middle cerebral artery responsiveness.

**Figure 2.**
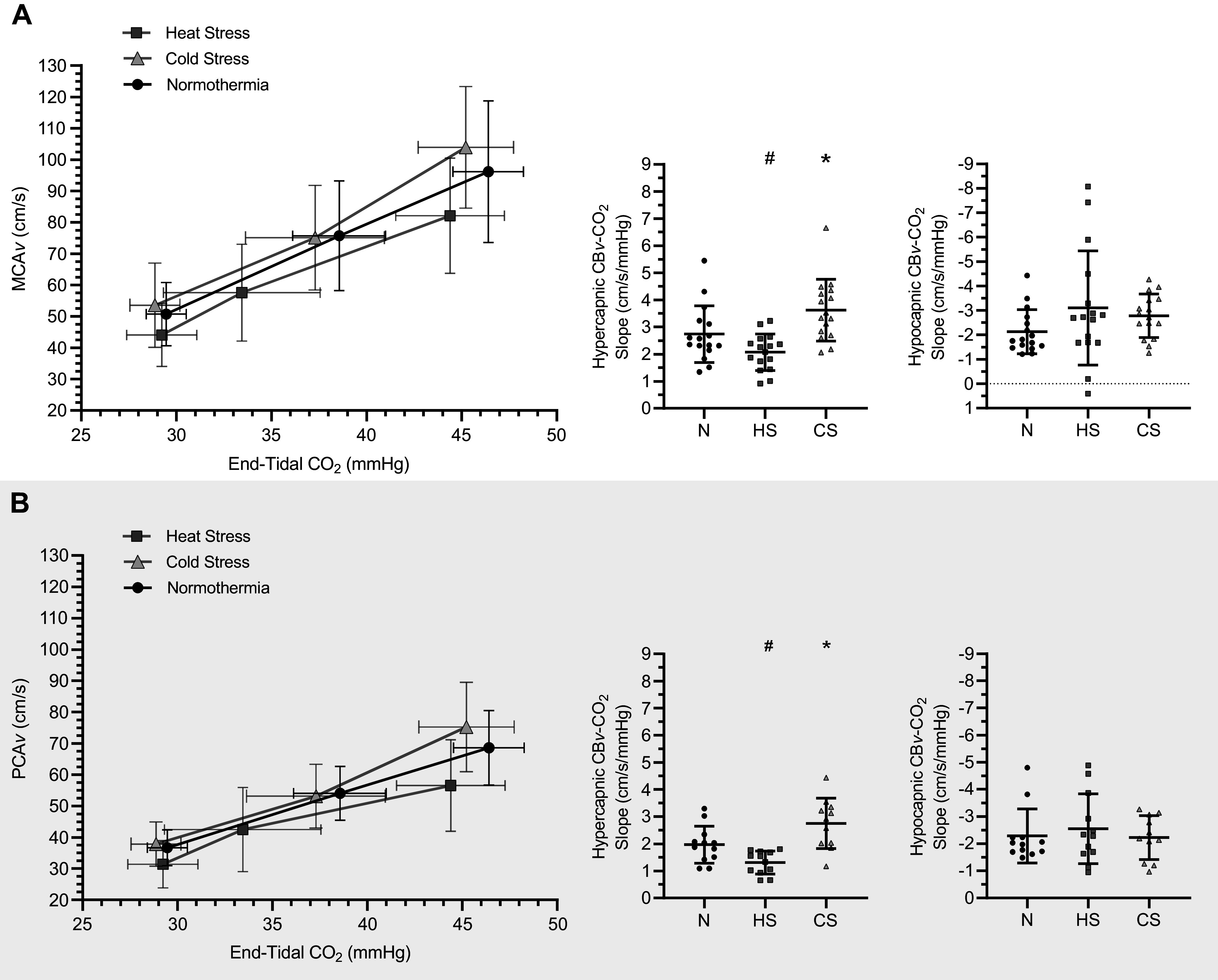
The middle and posterior cerebral artery blood velocity (MCA*v*, *A*; PCA*v*, *B*) slope at hyper- and hypocapnia (MCA*v*- and PCA*v*-CO_2_ responsiveness) during normothermic (N), passive heat stress (HS), and passive cold stress (CS) conditions. Data analyzed using a one-way repeated-measures ANOVA or mixed-effects model. #Significantly different to normothermia; *significantly different to heat stress.

MCA*v*-CO_2_ responsiveness to hypercapnia differed between thermal conditions [*F*(2,25) = 15.25, *P* < 0.001; Fig. 2 and Table 2]. MCA*v*-CO_2_ responsiveness to hypercapnia decreased during heat stress (−0.67 ± 0.89 cm/s/mmHg; *P* = 0.02) and increased during cold stress (+0.98 ± 1.33 cm/s/mmHg; *P* = 0.03) when compared with normothermia. MCA*v*-CO_2_ responsiveness to hypercapnia was lower during heat stress compared with cold stress (−1.65 ± 1.34 cm/s/mmHg; *P* < 0.001). MCA*v*-CO_2_ responsiveness to hypocapnia was similar between thermal conditions [*F*(1,20) = 0.33, *P* = 0.63]. Simple linear regression analysis of MCA*v*-CO_2_ responsiveness found similar slopes (*P* = 0.43) but differing *y*-intercepts (*P* = 0.003), with cold stress having a higher *y*-intercept compared with both normothermia and heat stress (normothermia, *y* = 2.68*x* − 28.06; heat stress, *y* = 2.42*x* − 24.37; cold stress, *y* = 3.08*x* − 36.66).

**Table 2.** Cerebrovascular-CO_2_ responsiveness values for the middle cerebral artery (MCAv-CO_2_ responsiveness; n = 16) and posterior cerebral artery (PCAv-CO_2_ responsiveness; n = 12) during normothermia, heat stress, and cold stress

	Normothermia	Heat Stress	Cold Stress
MCA*v*-CO_2_ responsiveness, cm/s/mmHg			
Hypercapnia	2.74 ± 1.05	2.07 ± 0.67#	3.72 ± 1.07#*
Hypocapnia	−2.88 ± 1.44	−3.11 ± 2.34	−2.71 ± 0.96
MCA*v*-CVC-CO_2_ responsiveness, cm/s/mmHg/mmHg			
Hypercapnia	0.02 ± 0.01	0.02 ± 0.01	0.03 ± 0.01
Hypocapnia	−0.04 ± 0.01	−0.08 ± 0.08	−0.03 ± 0.01*
PCA*v*-CO_2_ responsiveness, cm/s/mmHg			
Hypercapnia	1.96 ± 0.66	1.31 ± 0.43#	2.85 ± 0.80#*
Hypocapnia	−2.29 ± 1.00	−2.56 ± 1.28	−2.07 ± 0.80
PCA*v*-CVC-CO_2_ responsiveness, cm/s/mmHg/mmHg			
Hypercapnia	0.02 ± 0.01	0.01 ± 0.01	0.02 ± 0.01
Hypocapnia	−0.03 ± 0.01	−0.05 ± 0.04	−0.02 ± 0.01*

Values are represented as means ± SD; *n* − 1 participant for PCA outcome variables in cold stress condition. Data analyzed using a one-way repeated-measures ANOVA or mixed-effects model. #Significantly different from normothermia; *significantly different from heat stress. CVC, cerebrovascular conductance; MCA, middle cerebral artery; PCA, posterior cerebral artery.

MCA*v*-CVC-CO_2_ responsiveness to hypercapnia was similar between thermal conditions [*F*(2,30) = 2.22, *P* = 0.13]. However, MCA*v*-CVC-CO_2_ responsiveness to hypocapnia differed between thermal conditions [*F*(1,16) = 6.00, *P* = 0.03], remaining unchanged (i.e., *P* > 0.05) between heat stress and normothermia (*P* = 0.09), and cold stress and normothermia (*P* = 0.06), but elevated during heat stress when compared with cold stress (+0.05 ± 0.08 cm/s/mmHg/mmHg; *P* = 0.04).

#### Posterior cerebral artery responsiveness.

PCA*v*-CO_2_ responsiveness to hypercapnia differed between thermal conditions [*F*(2,17) = 20.10, *P* < 0.001; Fig. 2 and Table 2]. During heat stress, PCA*v*-CO_2_ responsiveness to hypercapnia decreased when compared with normothermia (−0.64 ± 0.62 cm/s/mmHg; *P* = 0.01) but was elevated during cold stress when compared with normothermia (+1.00 ± 0.82 cm/s/mmHg; *P* = 0.01). PCA*v*-CO_2_ responsiveness to hypercapnia decreased in heat stress when compared with cold stress (−1.57 ± 1.00 cm/s/mmHg; *P* = 0.001). PCA*v*-CO_2_ responsiveness to hypocapnia was similar between thermal conditions [*F*(2,16) = 1.02, *P* = 0.36]. Simple linear regression analysis of PCA*v*-CO_2_ responsiveness found similar slopes (*P* = 0.21) but differing *y*-intercepts (*P* = 0.003), with cold stress having a higher *y*-intercept compared with both normothermia and heat stress (normothermia, *y* = 1.89*x* − 19.27; heat stress, *y* = 1.58*x* − 12.98; cold stress, *y* = 2.29*x* − 29.42).

PCA*v*-CVC-CO_2_ responsiveness to hypercapnia was similar between thermal conditions [*F*(2,19) = 3.03, *P* = 0.08], whereas PCA*v*-CVC-CO_2_ responsiveness to hypocapnia differed between conditions [*F*(1,12) = 4.96, *P* = 0.04; Table 2]. PCA*v*-CVC-CO_2_ responsiveness to hypocapnia was similar during heat stress (*P* = 0.16) and cold stress (*P* = 0.12) when compared with normothermia but increased during heat stress when compared with cold stress (+0.02 ± 0.02 cm/s/mmHg/mmHg; *P* = 0.002).

### Cerebrovascular-CO_2_ Responsiveness in the Anterior and Posterior Circulation

During normothermia, CB*v*-CO_2_ responsiveness to hypercapnia was similar between the MCA and PCA [*t*(11) = 1.20, *P* = 0.25]. However, CB*v*-CO_2_ responsiveness to hypocapnia was greater in the PCA when compared with the MCA [+0.6 ± 0.7%CBv/mmHg; *t*(11) = 2.88, *P* = 0.02; Table 3]. Similarly, in normothermia, CB*v*-CVC-CO_2_ responsiveness to hypercapnia was similar between vessels [*t*(11) = 1.39, *P* = 0.19], whereas CB*v*-CVC-CO_2_ responsiveness to hypocapnia was greater in the PCA when compared with the MCA [+0.7 ± 0.7%CB*v*/mmHg/mmHg; *t*(11) = 2.82, *P* = 0.02].

**Table 3.** Relative cerebrovascular-CO_2_ responsiveness (CBv-CO_2_ responsiveness; %CBv/mmHg) and cerebrovascular conductance-CO_2_ responsiveness (CBv-CVC-CO_2_ responsiveness; %CBv/mmHg/mmHg) values for the middle cerebral artery (MCA) and posterior cerebral artery (PCA) during normothermia, heat stress, and cold stress conditions (n = 12)

	Normothermia	Heat Stress	Cold Stress
CBv-CO_2_ responsiveness to hypercapnia, %CBv/mmHg			
MCA	3.9 ± 0.9	3.5 ± 1.5	5.2 ± 1.3
PCA	3.7 ± 1.2	3.3 ± 1.3	5.1 ± 1.3
CBv-CVC-CO_2_ responsiveness to hypercapnia, %CBv/mmHg/mmHg			
MCA	2.8 ± 0.9	2.7 ± 1.8	3.9 ± 1.6
PCA	2.5 ± 1.1	2.5 ± 1.4	3.9 ± 2.0
CBv-CO_2_ responsiveness to hypocapnia, %CBv/mmHg			
MCA	3.6 ± 1.2	5.4 ± 3.5	3.7 ± 1.3
PCA	4.2 ± 1.2*	6.3 ± 3.5	4.3 ± 1.8
CBv-CVC-CO_2_ responsiveness to hypocapnia, %CBv/mmHg/mmHg			
MCA	3.5 ± 1.2	5.6 ± 3.0	3.8 ± 1.2
PCA	4.2 ± 1.2*	5.9 ± 1.9	4.2 ± 1.4

Values are represented as means ± SD. Data analyzed using paired *t*-tests. *Significantly different to MCA. *n* − 1 participant for cold stress condition.

During passive heat and passive cold stress, CB*v*-CO_2_ responsiveness to hypercapnia [Heat: *t*(11) = 0.72, *P* = 0.49; Cold: *t*(10) = 0.19, *P* = 0.85] and hypocapnia [Heat: *t*(11) = 0.82, *P* = 0.43; Cold: *t*(10) = 1.68, *P* = 0.12] remained similar between the MCA and PCA. Similarly, CB*v*-CVC-CO_2_ responsiveness to hypercapnia [Heat: *t*(11) = 1.01, *P* = 0.49; Cold: *t*(10) = 0.06, *P* = 0.95] and hypocapnia [Heat: *t*(11) = 0.45, *P* = 0.66; Cold: *t*(10) = 1.24, *P* = 0.25] remained similar between the MCA and PCA during passive heat and cold stress.

## DISCUSSION

The main findings of this study were that *1*) CB*v*-CVC-CO_2_ responsiveness to hypocapnia was greater during heat stress when compared with cold stress; *2*) thermal stress-related changes in CB*v*-CO_2_ responsiveness to hypercapnia were driven by changes in MAP since conductance responsiveness was similar across conditions; and *3*) CB*v*-CO_2_ responsiveness to hypocapnia was greater in the PCA compared with the MCA in normothermic, but not heat or cold stress, conditions. Collectively, these findings indicate that during heat stress there is a greater cerebrovascular sensitivity to hypocapnia (i.e., a greater decrease in CB*v* per mmHg decrease in end-tidal CO_2_) and that thermal stress-induced changes in mean arterial pressure influence the cerebrovascular response to hypercapnia. In addition, regional differences in cerebrovascular-CO_2_ responsiveness exist within the intracranial arteries during normothermia.

### Effect of Thermal Stress on Cerebrovascular-CO_2_ Responsiveness

In contrast to our hypothesis, the present study showed that CB*v*-CO_2_ responsiveness to hypercapnia in both the MCA and PCA was lower during heat stress when compared with normothermia. This differs from previous findings that MCA*v*-CO_2_ responsiveness to hypercapnia during passive heating either increases (8) or remains unchanged (4, 10, 11) when compared with normothermia. Methodological differences [e.g., severity of the heat stimulus, order of the CO_2_ challenges blunting the hypercapnic response (12)] may account for the differences in these previous findings to that of the present study. Of note, Low et al. (11) only reported the slope of the MCA*v*-CO_2_ relationship as a measure of conductance (i.e., MCA*v*-CVC-CO_2_ responsiveness), seeing no change between heat stress and normothermia. Similarly, CB*v*-CVC-CO_2_ responsiveness to hypercapnia in the present study remained unchanged, indicating that heat stress-related decreases in MAP appear to determine changes in CB*v* responsiveness to hypercapnia rather than a change in vascular sensitivity to CO_2_ itself. During cold stress, CB*v*-CO_2_ responsiveness to hypercapnia increased when compared with both normothermia and heat stress conditions. To the best of our knowledge, this is the first study to examine the effect of passive cold stress on CB*v*-CO_2_ responsiveness in the hypercapnic range. Since CB*v*-CVC-CO_2_ responsiveness to hypercapnia was similar between passive cold stress, heat stress, and normothermia, the cold stress-related increase in MAP also appears to determine increases in CB*v*-CO_2_ responsiveness to hypercapnia. Therefore, changes in CB*v*-CO_2_ responsiveness to hypercapnia across thermal conditions appear to be driven by changes in perfusion pressure rather than a change in vascular CO_2_ sensitivity.

The present study showed similar CB*v*-CO_2_ responsiveness to hypocapnia between normothermic, cold stress, and heat stress conditions. Only one study has examined CB*v*-CO_2_ responsiveness to hypocapnia during cold exposure, showing a tendency for increased responsiveness during cold-water immersion (−1°C in T_C_) when compared with normothermia [*P* = 0.05 (9)], with the difference between these findings and the present study likely due to the mode and magnitude of the cold stress stimulus. When changes in MAP are considered, CB*v*-CVC-CO_2_ responsiveness to hypocapnia was greater during heat stress compared with cold stress, indicating that during heat stress, there is a greater sensitivity in the CB*v* response to changing CO_2_ (i.e., a greater decrease in CB*v* per mmHg decrease in CO_2_). Notably, in the current study, a heat stress-induced decrease in resting CB*v* and end-tidal CO_2_ shifted the operating point of the CB*v*-CO_2_ relationship. This appeared to reduce the range by which further decreases in CB*v* can occur in response to hypocapnia before, for example, a loss of consciousness. Although not statistically significant (*P* = 0.06), MCA*v*-CVC-CO_2_ responsiveness to hypocapnia was, on average, lower during cold stress compared with normothermia suggesting a blunted sensitivity of the CB*v* response to decreased CO_2_. This may indicate preservation of CB*v* during cold exposure; however, further research is needed to consolidate this finding. Overall, we report that heat and cold stress differentially affect CB*v*-CVC responsiveness to hypocapnia, with a greater responsiveness during heat stress exacerbating an already challenged CB*v*, whereas a reduced responsiveness in cold stress will aid the maintenance of cerebral perfusion.

### Regional Differences in Cerebrovascular-CO_2_ Responsiveness

In contrast to our hypothesis, the present study found MCA*v*- and PCA*v*-CO_2_ responsiveness to be similar during thermal stress (both heat and cold stress). Heat stress-induced regional differences in cerebrovascular-CO_2_ responsiveness have been reported previously, with an enhanced responsiveness in the external carotid artery (ECA) and reduced responsiveness in the internal carotid artery (ICA) (10). Since the ECA mainly supplies the cutaneous circulation, increased ECA-CO_2_ responsiveness during heat stress likely acts as a thermoregulatory mechanism to aid heat dissipation, and ICA-CO_2_ responsiveness decreases to aid CBF maintenance during heat stress (19). Although heat stress has been shown to cause a similar relative decrease in MCA*v* and PCA*v* (5), cerebrovascular-CO_2_ responsiveness has not previously been compared in these vessels under heat or cold stress conditions. Our results indicate no regional prioritization of CB*v* via changing CO_2_ responsiveness in the anterior and posterior circulations when the MCA and PCA are used to represent these circulations. Regional cerebral blood flow may still be differentially distributed by thermal stress but through other regulatory mechanisms (e.g., cerebral autoregulation). Further research is needed to better understand the regional prioritization of CBF and how this may cause or impact adverse events during exposure to extreme thermal environments.

In normothermia, the PCA had a greater CB*v*-CO_2_ responsiveness to hypocapnia when compared with the MCA. Cerebrovascular-CO_2_ responsiveness to hypocapnia has previously been reported as similar between the MCA and PCA (17), although over a much wider hypocapnic range (15, 20, and 30 mmHg). Of note, during severe hypocapnia (∼25 mmHg and lower), the cerebrovasculature is thought to reach a point of maximal constriction (20), where changes in $\text{P}{\text{a}}_{{\text{CO}}_{2}}$ would not be represented by further changes in CB*v*. Subsequently, relative changes in cerebrovascular-CO_2_ responsiveness in the MCA and PCA may be similar at lower end-tidal CO_2_ concentrations (e.g., 15 and 20 mmHg) when the limit of the vessel caliber has been reached. The present study reports differences in MCA*v*- and PCA*v*-CO_2_ responsiveness to one milder hypocapnic stage (30 mmHg) and, therefore, within the operating range of the vascular responsiveness. Of note, this normothermic difference in MCA*v*- and PCA*v*-CO_2_ responsiveness was in contrast to the similar responsiveness between anterior and posterior circulations when under thermal stress (as discussed above). Overall, the present study indicates that CBF regulation is better preserved in the anterior circulation during normothermic hypocapnia.

### Effect of Thermal Stress at Rest

The present findings are consistent with previous studies where hyperthermia-induced hyperventilation and subsequent hypocapnia (21, 22) corresponded to a reduced resting CB*v* during passive heating (4, 23). However, this observed decrease in CB*v* was greater than that seen previously with a similar heat stress perturbation (4). Although hypocapnia is the predominant cause of a reduced CB*v* during passive heating (3, 7), previous work has shown that even with the restoration of end-tidal CO_2_ to a eucapnic level during heat stress, it only partially restores CB*v* (6, 24). Thus, the modulation of CB*v* with passive heating is likely due to a combination of changes in end-tidal CO_2_ concentrations, perfusion pressure, and other regulatory factors. Further research is needed to fully elucidate the underlying mechanisms behind the reduction in CB*v* during heat stress. Importantly, the decrease in CB*v* reported here illustrates a reduced cerebrovascular reserve to protect against further decreases in cerebral perfusion before orthostatic intolerance and syncope events are experienced during passive heat exposure.

The physiological response to passive cold stress is less well documented. The present study supports previous findings that CB*v* remains unchanged (15) and ventilation increases (25) with passive cold stress. This is likely driven by the metabolic demands of shivering, whereby changes in ventilation are primarily caused by increased tidal volume (26). This, and the unchanged respiratory frequency, may account for the similar end-tidal CO_2_ observed between cold stress and normothermia. Cold stress significantly increased MAP, likely a consequence of redistribution of blood away from the peripheral circulation to aid heat preservation (27). Yet, CB*v* remained similar to values seen in normothermia, with CB*v* conductance lower during cold stress as compared with normothermia and heat stress conditions. This may be explained by other CBF regulatory mechanisms, particularly cerebral autoregulation, which is recognized as being more effective at compensating for transient hypertension than hypotension (28, 29). Overall, passive cold stress and heat stress result in distinctive ventilatory, cardiovascular, and cerebrovascular responses, which interact in an integrative manner. The CB*v* response to both thermal conditions appears to be largely driven by thermally mediated changes in MAP but with changes in MAP helping to maintain CB*v* during cold stress but challenging CB*v* during heat stress.

### Perspectives

Cerebrovascular responsiveness to CO_2_ has been utilized as a clinical tool to provide a measure of cerebrovascular function or dysfunction, as well as predict the risk of future cerebrovascular disease (30–32). A greater change (either increase or decrease) in cerebral blood flow to hyper- or hypocapnia is seen as indicative of a greater cerebrovascular reserve capacity and thus improved cerebrovascular health (2). The present study reports a greater CB*v*-CVC-CO_2_ responsiveness to hypocapnia during heat stress compared with cold stress, an outcome that would be seen as beneficial in clinical settings but is detrimental to the maintenance of cerebral perfusion during heat exposure. This disparity between what is deemed an improved or beneficial response arises when functional outcomes such as cerebrovascular-CO_2_ responsiveness are examined under markedly different contexts (i.e., chronic clinical contexts used to assess or predict disease vs. acute responses to stimuli within a healthy model). Care should be taken when comparing reported cerebrovascular responsiveness outcomes from studies in different contexts, as acute changes in cerebrovascular responsiveness may not necessarily have the same meaning for all populations in all contexts.

### Considerations/Study Limitations

Doppler ultrasound was used to measure blood velocity in the MCA and PCA. A primary assumption of TCD is that the insonated vessel maintains a constant diameter. Although this assumption has been reported as valid (33, 34), more recent MRI studies have reported changing vessel diameters in response to changing CO_2_ (35, 36), which may result in possible over- and underestimations of CBF when CB*v* is used as an index of absolute flow. Despite this, assessment of cerebrovascular responsiveness by TCD has been shown to offer valuable information on cerebrovascular function, provided data are interpreted with these limitations in mind (37).

The present study used a steady-state CO_2_ technique to measure cerebrovascular-CO_2_ responsiveness. This technique was chosen as it incorporates both the ventilatory and cerebrovascular response to a steady-state CO_2_ stimulus, providing a more physiologically relevant approach. The use of different techniques to measure cerebrovascular- or ventilatory-CO_2_ responsiveness (i.e., steady state or rebreathing) may elicit different outcomes as they stimulate physiological systems in a different manner. For example, a rebreathing technique abolishes the Pco_2_ gradient throughout the body (e.g., between end-tidal and arterial concentrations) and, therefore, measures only the ventilatory response unaffected by the cerebrovascular response (2). As such, the present findings should be considered with the methodology used in mind, and that outcomes may differ if a different technique is used.

Within the literature, “passive cold stress” can refer to several distinct modes of cooling. Use of air exposure, water immersion, or a water perfusion suit to elicit cold stress is likely to result in different cerebrovascular-CO_2_ responsiveness outcomes. Cold-water immersion in particular is known to cause changes in hydrostatic pressure, leading to centralization of blood within the body and altering cardiovascular variables [e.g., cardiac output (38)]. In the present study, we use a water perfusion suit to induce cold stress, which does not induce the same changes in hydrostatic pressure, and consequently, the present results may not be directly comparable with studies that use other modes of cooling (e.g., water immersion).

Both male and female participants were included in this study, with females tested during the early follicular phase of their menstrual cycle. Sex differences, and differences within females across menstrual phases, in cerebrovascular-CO_2_ responsiveness have been demonstrated previously (39). In addition, elevated estrogen and progesterone during the menstrual cycle are associated with an elevation in core temperature alongside a shift in thermoregulatory responses to a higher core temperature [e.g., onset of cutaneous vasodilation and vasoconstriction, sweating, and shivering (40)]. Although unknown at present, it should be considered that differences may exist in the cerebrovascular response to CO_2_ during thermal stress for females when tested during ovulatory or mid-luteal phases when estrogen and progesterone concentrations are higher. Furthermore, though the present study has a balanced representation of males and females, the large individual variation observed in the current sample indicates that a larger sample size would be required to adequately investigate sex differences in cerebrovascular-CO_2_ responsiveness during thermal stress.

Changes in end-tidal CO_2_ were used to calculate cerebrovascular-CO_2_ responsiveness, based on the assumption that end-tidal CO_2_ accurately represents arterial CO_2_. Although these two variables can differ with metabolic CO_2_ production and tidal volume, they do not differ by changes in breathing frequency (41) and have been validated across a range of core temperatures (42).

An a priori power analysis was not performed in the current study. However, a post hoc sensitivity analysis has been conducted to inform what effect sizes the reported sample size is able to detect. A repeated-measures one-way ANOVA with 16 participants would be sensitive to effects of η^2^ = 0.10 with 80% power (α = 0.05) and as such would not be able to reliably detect effects smaller than η^2^ = 0.10 [a moderate effect size (43)]. Effect sizes for nonsignificant results in the current study ranged from η^2^ = 0.02 to η^2^ = 0.13 (calculated using the reported *F* value and associated degrees of freedom), and subsequently, this should be considered when interpreting the results.

### Summary

The findings from this study show that heat stress poses a multifaceted challenge to the maintenance of cerebral perfusion. Heat stress-induced reductions in MAP and end-tidal CO_2_ reduce the cerebrovascular reserve capacity, which is further impaired by a greater cerebrovascular responsiveness to hypocapnia. In addition, this study reports the effect of passive cold stress on cerebrovascular responsiveness across the CO_2_ range for the first time. CB*v*-CO_2_ sensitivity during cold stress was shown to be unchanged compared with normothermia, but an increased MAP resulted in a greater CB*v* response to hypercapnia. Overall, exposure to passive heat and passive cold stress results in distinct changes in perfusion pressure that in turn impact the regulation of cerebral blood velocity. However, only heat stress presents a challenge to the maintenance of cerebral perfusion, with lower perfusion pressure and greater cerebrovascular responsiveness to hypocapnia likely contributing to the occurrence of adverse events (e.g., syncope) during heat exposure.

## DATA AVAILABILITY

The data that support the findings of this study are available from the corresponding author upon reasonable request.

## SUPPLEMENTAL DATA

10.6084/m9.figshare.24442489 Supplemental Table S1: https://doi.org/10.6084/m9.figshare.24442489.

## GRANTS

This research was funded by the University of Birmingham.

## DISCLOSURES

No conflicts of interest, financial or otherwise, are declared by the authors.

## AUTHOR CONTRIBUTIONS

R.A.I.L. and S.J.E.L. conceived and designed research; B.D.S., R.A.I.L., and S.J.E.L. performed experiments; B.D.S. analyzed data; B.D.S., R.A.I.L., and S.J.E.L. interpreted results of experiments; B.D.S. prepared figures; B.D.S., R.A.I.L., and S.J.E.L. drafted manuscript; B.D.S., R.A.I.L., and S.J.E.L. edited and revised manuscript; B.D.S., R.A.I.L., and S.J.E.L. approved final version of manuscript.
